# Deficiência de Testosterona em Homens Hipertensos: Prevalência e Fatores Associados

**DOI:** 10.36660/abc.20230138

**Published:** 2024-03-21

**Authors:** Leandra Analia Freitas Negretto, Nelson Rassi, Leonardo Ribeiro Soares, Amanda Bueno Carvalho Saraiva, Maria Emília Figueiredo Teixeira, Luciana da Ressurreição Santos, Ana Luiza Lima Souza, Paulo Cesar B. Veiga Jardim, Weimar Kunz Sebba Barroso de Souza, Thiago de Souza Veiga Jardim

**Affiliations:** 1 Universidade Federal de Goiás – Liga de Hipertensão Arterial Goiânia GO Brasil Universidade Federal de Goiás – Liga de Hipertensão Arterial, Goiânia, GO – Brasil; 2 Hospital Geral de Goiânia – Dr. Alberto Rassi Goiânia GO Brasil Hospital Geral de Goiânia – Dr. Alberto Rassi, Goiânia, GO – Brasil

**Keywords:** Testosterona, Hipertensão, Obesidade, Hipogonadismo

## Abstract

**Fundamento:**

A deficiência de testosterona (DT) é uma condição prevalente em nosso meio e ainda muito negligenciada. A hipertensão arterial (HA) é um de seus possíveis fatores associados.

**Objetivos:**

Determinar a prevalência de DT em uma população masculina hipertensa e os fatores associados à sua ocorrência, como idade, tempo de diagnóstico de HA, número de classes de anti-hipertensivos, índice de massa corporal (IMC), diabetes, dislipidemia, doença renal crônica (DRC), sintomas positivos de DT (questionário ADAM positivo) e uso de espironolactona.

**Métodos:**

Estudo transversal com aplicação do questionário ADAM, e avaliação de dados bioquímicos, clínicos e antropométricos. Os pacientes foram estratificados em grupos de DT e testosterona normal. As variáveis categóricas foram comparadas pelo teste do qui-quadrado e as variáveis contínuas pelo teste de Mann-Witney; as variáveis com significância (p<0,05) foram submetidas à regressão linear multivariada.

**Resultados:**

A prevalência de DT foi de 26,8%. Houve associação entre DT e IMC (p=0,0007), mas não houve com idade (p=0,0520), tempo de diagnóstico de HA (p=0,1418), número de classes de anti-hipertensivos (p=0,0732), diabetes (p=0,1112); dislipidemia (p=0,3888); presença de DRC (p=0,3321); uso de espironolactona (p=0,3546) e questionário ADAM positivo (p=0,2483).

**Conclusões:**

A prevalência de DT foi alta e houve associação positiva com IMC. A testosterona total (TT) declinou 8,44 ng/dL com o aumento de 1 kg/m^2^ no IMC e caiu 3,79 ng/dL com o avanço em um ano na idade.

**Figure:**
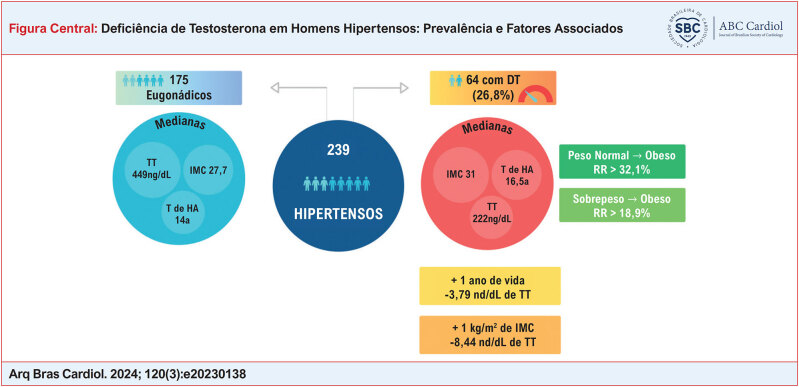


## Introdução

A deficiência de testosterona (DT) é resultante da falha testicular em produzir níveis fisiológicos de testosterona (T) devido à ruptura de um ou mais elementos do eixo hipotálamo-hipófise-testículo (HHT). Quando a DT é confirmada e está associada a sinais clínicos de deficiência androgênica, configura-se o hipogonadismo masculino.^1^ A prevalência de hipogonadismo, na população geral masculina, varia com a idade, sendo de seis a 12,3% entre 40 e 69 anos, respectivamente.^2^

A DT pode causar diversos sinais e sintomas, que dependem da idade de apresentação e de sua gravidade,^3^ como: diminuição da libido, disfunção erétil, diminuição do volume de ejaculação, perda de pelos corporais e faciais, fraqueza, diminuição da densidade óssea, diminuição da massa magra, aumento da gordura corporal, fadiga e anemia.^3 , 4^ Além disso, a sobrevida de homens com baixos níveis de T é inferior à de homens com valores normais de T (eugonadismo), sendo a mortalidade por todas as causas de 34,9% *vs* . 20,1%, respectivamente.^5^

A hipertensão arterial (HA) foi associada à DT em alguns estudos, como no estudo *"Hypogonadism in males"* (HIM),^6^ no qual foi observado que uma proporção mais alta de pacientes com DT era hipertensa em comparaçao a indivíduos com níveis normais do hormônio. Segundo Svartberg et al.,7 indivíduos hipertensos terão valores totais de T menores que os não hipertensos, independente da idade. Já Smith et al.,8 mostraram que a privação androgênica em homens em tratamento contra câncer de próstata poderia induzir HA.

A relação entre HA e DT ainda possui diversas lacunas, incluindo aspectos epidemiológicos, diagnósticos e terapêuticos. Nesse contexto, o objetivo do presente estudo foi determinar a prevalência de DT em uma população brasileira de homens hipertensos acompanhados em serviço de referência no tratamento da HA. Ainda, o estudo avaliou, nessa população, fatores associados à sua ocorrência – idade, tempo de diagnóstico de HA, número de classes de anti-hipertensivos utilizados, índice de massa corporal (IMC),^9^ presença de diabetes mellitus (DM),^10^ dislipidemia (DLP),^11^ doença renal crônica (DRC),^12^ sintomas de deficiência androgênica (segundo o questionário ADAM, *Androgen Deficiency, in the Aging Male"* ^13^) e uso de espironolactona.

## Métodos

### Desenho do estudo

Estudo transversal realizado em serviço de referência no tratamento da HA na região central do Brasil. Os dados foram coletados pela equipe assistente durante consultas ambulatoriais.

### Participantes

Para serem incluídos, os pacientes deveriam ter idade entre 18 e 85 anos, ser do sexo masculino, e comparecer às consultas ambulatoriais ao menos uma vez ao ano. Foram excluídos pacientes que não realizaram os exames solicitados, transsexuais, e pacientes que apresentavam déficits cognitivos que os incapacitassem a responder ao questionário ADAM.^13^

O cálculo amostral foi utilizando o G*Power 3,1, adotando prevalência esperada de 12%,^2^ intervalo de confiança (IC) de 95%, nível de significância de 0,05 e 80% de poder do teste, resultando no tamanho mínimo de 148 indivíduos.

### Procedimentos de coleta de dados

Nesta pesquisa, foram consideradas as variáveis: idade, tempo de diagnóstico de HA, número de classes de anti-hipertensivos usados, uso de espironolactona, IMC,^9^ presença DM,^10^ DLP,^11^ DRC,^12^ além de sintomas positivos de deficiência androgênica (questionário ADAM^13^).

Durante consultas ambulatoriais, após a assinatura do termo de consentimento livre e esclarecido (TCLE), foram aferidos peso e altura, pesquisadas as variáveis mencionadas e solicitados exames incluindo dosagem de T total (TT). Para definir DT o ponto de corte de TT foi menor que 300 ng/dL (12nmol/L), conforme as recomendações da *Endocrine Society* ^1^ e da *American Urological Association* .^14^ As amostras de sangue foram coletadas em jejum, pela manhã, e os exames foram realizados por laboratórios randômicos que utilizam a metodologia dos imunoensaios.15

O questionário ADAM, da Universidade de Saint Louis, consiste em 10 perguntas sobre sintomas de deficiência androgênica do tipo "sim ou não".^13^ Esse questionário apresenta sensibilidade de 88% e especificidade de 60%,^16^ sendo o seu emprego recomendando como instrumento de triagem de hipogonadismo.^17^

### Análise estatística

Foram avaliados 239 participantes e estratificados em dois grupos segundo o *status* de TT em: (1) indivíduos com DT ( *n* =64; 26,8%) e (2) eugonádicos ( *n* =175; 73,2%). Na estatística descritiva, foram calculadas, para as variáveis categóricas: as frequências absolutas ( *n* ) e relativas percentuais [ *f(%)* ]; e para as variáveis contínuas: mediana e intervalo interquartil (IIQ). As variáveis categóricas foram: *status* de TT; idade; tempo de HA; número de classes de anti-hipertensivos; IMC; DM; DLP; DRC; uso de espironolactona e questionário ADAM. As variáveis quantitativas contínuas foram: idade; tempo de HA; número de classes de anti-hipertensivos; IMC; e nível de TT (em ng/dL).

Para a estatística inferencial, as variáveis categóricas, estratificadas pelo *status* de TT, foram comparadas pelos testes: qui-quadrado, exato de Fisher ou G. Para a variável com significância estatística, foram calculados Odds Ratio (OR) e IC de 95% para avaliar as chances de apresentar DT entre as categorias.

Para as variáveis contínuas, foram calculadas a normalidade dos dados com o teste de Kolmogorov-Smirnov e a homogeneidade de variância, pelo teste de Levene. Todas as variáveis contínuas apresentaram distribuição não paramétrica sem homogeneidade (p<0,05). Desta forma, foi aplicado o teste de Mann-Whitney para amostras independentes. Posteriormente, para as variáveis com p<0,20, foi aplicado teste de regressão linear multivariada. Finalmente, foi aplicado o teste de correlação de Spearman.^18^

## Resultados

Foram recrutados 276 homens hipertensos; 37 participantes foram excluídos – 18 apresentavam idade superior a 85 anos, 11 se recusaram a participar do estudo, três apresentavam déficit cognitivo, dois tinham esquizofrenia, dois faleceram antes da coleta de sangue para medida do TT e um era transexual. Entre os 239 participantes incluídos, 175 (73,2%) apresentaram níveis normais de TT (eugonadismo) e 64 apresentaram níveis baixos de TT, determinando a prevalência de DT em 26,8%. Os resultados estão sintetizados na Figura Central.

A Tabela 1 apresenta as variáveis *status* de TT, ou seja, DT ou eugonádico, idade, tempo de diagnóstico de HA, número de classes de anti-hipertensivos, IMC,^9^ diagnóstico de DM,^10^ DLP,^11^ e DRC,^12^ uso de espironolactona e questionário ADAM.^13^ Esses dados estão apresentados como frequência absoluta *(n)* e relativa percentual [ *f(%)* ].

**Tabela 1:** Estatística descritiva das variáveis categóricas, expressas como frequência absoluta ( *n* ) e relativa percentual [ *f(%)* ], dos 239 participantes; Goiânia, Brasil, 2023

Variáveis (N=239)			n	f(%)
***Status* de Testosterona**				
	Deficiência de Testosterona		64	26,8
	Eugonádico		175	73,2
**Idade (anos)**				
	Até 60 anos		91	38,1
	60 anos ou mais		148	61,9
**Tempo de HA (anos)**				
	Até 15 anos		126	52,7
	Acima de 15 anos		97	40,6
	Sem Informação		16	6,7
**Número de classes de anti-hipertensivos**				
	Nenhum		8	3,3
	Até 3		184	77,0
	Acima de 3		47	19,7
**IMC (kg/m^2^)**				
	Normal (18,6 até 24,9)		51	21,3
	Sobrepeso (25,0 até 29,9)		100	41,8
	Obesidade (≥30,0)			
		Grau I (30,0 até 34,9)	58	24,3
		Grau II (35,0 até 39,0)	20	8,4
		Grau III (≥40,0)	8	3,3
	Sem Informação		2	0,8
**Diabetes**				
	Sim		78	32,6
	Não		161	67,4
**Dislipidemia**				
	Sim		172	72,0
	Não		67	28,0
**DRC**				
	Sim		78	32,6
	Não		161	67,4
**Espironolactona**				
	Sim		23	9,6
	Não		216	90,4
**Questionário ADAM**				
	Negativo		24	10,0
	Positivo		184	77,0
	Sem Informação		31	13,0

N: número total de indivíduos no grupo amostral; n: frequência absoluta; f(%): frequência relativa percentual; HA: hipertensão arterial; IMC: índice de massa corporal; DRC: doença renal crônica; Questionário ADAM (do inglês, *Androgen Deficiency, in the Aging Male* ).

A Tabela 2 compara as variáveis e suas categorias entre os grupos DT e eugonádicos. Foi observada diferença estatisticamente significativa nas categorias de IMC (p=0,0007) entre os grupos, sendo observado maior número de indivíduos com obesidade nos pacientes com DT.

**Tabela 2:** Estatística inferencial das variáveis categóricas, dos 239 participantes, estratificados pelo *status* de testosterona. Goiânia, Goiás, Brasil, 2023

Variáveis (N=239)			Déficit Testosterona (n=64; 26,8%)		Eugonádico (n=175; 73,2%)		p-valor *
			*n*	*f(%)*	*n*	*f(%)*	
**Idade (anos)**							
	Até 60 anos		18	28,1	73	41,7	
	60 anos ou mais		46	71,9	102	58,3	0,0520
**Tempo de HA (anos)**							
	Até 15 anos		28	43,8	98	56,0	
	Acima de 15 anos		30	46,9	67	38,3	0,1418
	Sem Informação		6	9,4	10	5,7	
**Número de classes de anti-hipertensivos**							
	Nenhum		0	0,0	8	4,6	
	Até 3		52	81,3	132	75,4	
	Acima de 3		12	18,8	35	20,0	0,0732
**IMC (kg/m^2^)**							
	Normal (18,6 até 24,9)		5	7,8	46	26,3	
	Sobrepeso(25,0 até 29,9)		23	35,9	77	44,0	
	Obesidade(≥30,0)						
		Grau I (30,0 até 34,9)	24	37,5	34	19,4	
		Grau II (35,0 até 39,0)	9	14,1	11	6,3	
		Grau III (≥40,0)	3	4,7	5	2,9	**0,0007**
	Sem Informação		0	0,0	2	1,1	
**Diabetes**							
	Sim		26	40,6	52	29,7	
	Não		38	59,4	123	70,3	0,1112
**Dislipidemia**							
	Sim		49	76,6	123	70,3	
	Não		15	23,4	52	29,7	0,3388
**DRC**							
	Sim		24	37,5	54	30,9	
	Não		40	62,5	121	69,1	0,3321
**Espironolactona**							
	Sim		8	12,5	15	8,6	
	Não		56	87,5	161	92,0	0,3546
**Questionário ADAM**							
	Negativo		4	6,3	20	11,4	
	Positivo		51	79,7	133	76,0	0,2483
	Sem Informação		9	14,1	22	12,6	

N: número total de indivíduos no grupo amostral; n: frequência absoluta; f(%): frequência relativa percentual; HA: hipertensão arterial; IMC: índice de massa corporal; DRC: doença renal crônica; Questionário ADAM (do inglês, Androgen Deficiency, in the Aging Male).

* teste do qui-quadrado; teste exato de Fisher ou teste G.

Houve predomínio de indivíduos com obesidade no grupo de DT, IMC (χ^2^=18,10; p=0,0001). Para a comparação entre as categorias de IMC, foram observadas diferenças estatisticamente significantes entre: IMC normal e obeso (OR=6,62; IC95%=2,39–18,32; p=0,0002), com acréscimo de risco relativo de 32,1%; e sobrepeso e obeso (OR=2,41; IC95%=1,28–4,54; p=0,0094), com acréscimo de risco relativo de 18,9%. A Tabela 3 mostra diferenças estatisticamente significativas entre os grupos DT e eugonádico nas variáveis "tempo de HA" (p=0,317), "IMC" (p<0,0001) e "TT" (p<0,0001).

**Tabela 3:** *Mediana* e intervalo interquartil ( *IIQ* ) das variáveis quantitativas contínuas de todos os 239 participantes e estratificadas e comparadas pelo status de testosterona. Goiânia, Goiás, Brasil, 2023

Variáveis	DT (n=64; 26,8%)			Eugonádico (n=175; 73,2%)			p-valor *	Total (N=239)		
	*Mediana*	*IIQ*		*Mediana*	*IIQ*			*Mediana*	*IIQ*	
		25%	75%		25%	75%			25%	75%
Idade (anos)	63,5	58,8	70,0	62,0	55,0	70,5	0,3476	63,0	55,5	70,0
Tempo de HA (anos)	**16,5**	8,0	24,3	**14,0**	6,0	20,0	**0,0317**	15,0	7,0	20,0
Número de anti-hipertensivos	3,0	2,0	3,0	3,0	2,0	3,0	0,7242	3,0	2,0	3,0
IMC (Kg/m^2^)	**31,0**	27,3	34,5	**27,7**	24,9	30,8	**<0,0001**	28,5	25,4	31,6
Testosterona total (ng/dL)	**220,0**	182,5	262,8	**449,0**	360,0	532,0	**<0,0001**	376,7	286,3	491,5

DT: deficiência de testosterona; N: número total de indivíduos no grupo amostral; Mediana: medida de tendência central; IIQ: intervalo interquartil de 25% e 75%; HA: hipertensão arterial; IMC: índice de massa corporal.

* Para gerar o p-valor, foi utilizado o teste de Mann-Whitney para amostras independentes.

A correlação de Spearman entre as variáveis quantitativas contínuas demonstrou que os valores de TT tiveram relação baixa, inversamente proporcional e estatisticamente significante com IMC (ρ=-0,26; p=0,0001), idade (ρ=-0,1760; p=0,0069) e tempo de diagnóstico de HA (ρ=-0,1410; p=0,0366) ( Tabela 4 ).

**Tabela 4:** Correlação de Spearman entre as variáveis quantitativas contínuas; Goiânia Brasil, 2023

Variáveis		Testosterona (ng/dL)	Idade (anos)	HA (anos)	Anti-hipertensivo	IMC (kg/m^2^)
Testosterona	ρ (rho)	1,0000				
	*p-valor*					
Idade (anos)	ρ (rho)	**-0,1760**	1,0000			
	*p-valor*	**0,0068**				
HA (anos)	ρ (rho)	**-0,1410**	**0,3530**	1,0000		
	*p-valor*	**0,0366**	**<0,0001**			
Anti-hipertensivo	ρ (rho)	-0,1046	**0,1700**	**0,2640**	1,0000	
	*p-valor*	0,1083	**0,0083**	**0,0001**		
IMC (kg/m^2^)	ρ (rho)	**-0,2600**	**-0,2330**	-0,0048	0,0521	1,0000
	*p-valor*	**0,0001**	**0,0003**	0,9434	0,4248	

HA: hipertensão arterial; IMC: índice de massa corporal.

**Figura 1:**
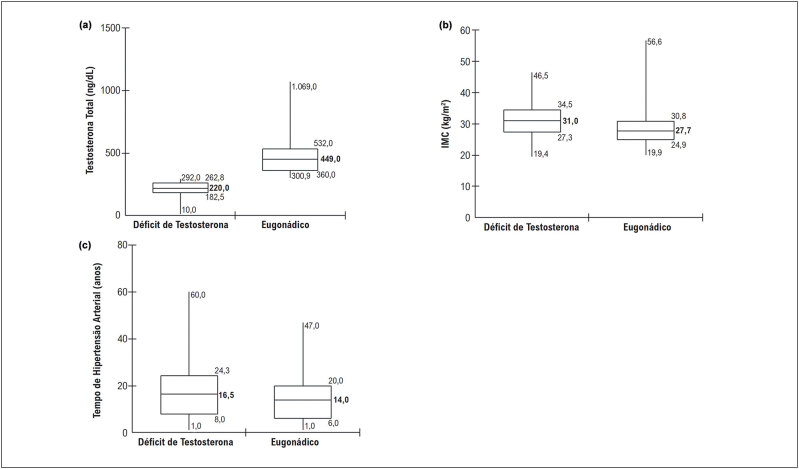
Gráficos de diagrama de caixa, com mediana, intervalo interquartil (25% e 75%), valores mínimo e máximo, das variáveis com diferença estatisticamente significante: (a) testosterona total; (b) IMC e (c) tempo de diagnóstico de hipertensão arterial. Goiânia, Goiás, Brasil.

A Figura 1 descreve as variáveis com diferença estatisticamente significante: (a) TT; (b) IMC e (c) tempo de diagnóstico de HA. Os indivíduos com DT apresentaram medianas de TT de 222,0 ng/dL, IMC 31,0 kg/m^2^ e 16,5 anos de diagnóstico de HA, enquanto os eugonádicos apresentaram medianas de TT de 449,0 ng/dL, IMC 27,7 Kg/m^2^ e 14,0 anos de diagnóstico de HA.

**Figura 2:**
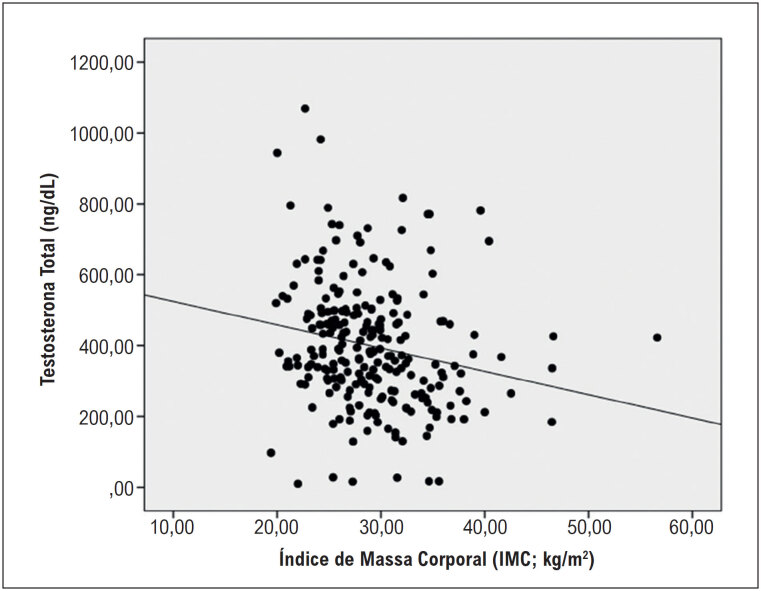
Relação entre nível de testosterona total (em ng/dL) e o índice de massa corporal (IMC, em kg/m^2^), com todos os participantes (N=239).

Foi realizada regressão linear multivariada com as variáveis com p-valores baixos (p<0,20), haja vista que todos os pressupostos estatísticos (linearidade, independência, homoscedasticidade, normalidade dos resíduos, não colinearidade e ausência de outliers) foram observados. As variáveis que mais influenciaram a TT foram: o IMC e a idade. O acréscimo de um ano de idade gerou decréscimo de 3,79 ng/dL na TT ( *B* =-3,79; IC95%=-5,57 até -2,06; p<0,0001) e o aumento de um kg/m^2^ no IMC gerou diminuição de 8,44 ng/dL na TT ( *B* =-8,44; IC95%= -12,57 até -4,32; p<0,0001) ( Figura 2 ).

## Discussão

Ao nosso conhecimento, este é o primeiro estudo que avaliou a prevalência de DT em uma população especificamente hipertensa. Encontramos a prevalência de DT de 26,8%, e observou-se associação de DT com o IMC (p=0,0007).

A obesidade é a condição clínica mais fortemente associada à diminuição das concentrações de T entre os homens.^19 , 20^ O excesso de peso promove supressão do eixo HHT de caráter funcional e potencialmente reversível.19 Baixos níveis de T levam ao crescimento da adiposidade devido à ausência do sinal inibidor na adipogênese e na captação lipídica, mediado pela enzima lipoproteína lipase, sobretudo em depósitos abdominais viscerais.^21^ Por outro lado, a perda de peso pode levar à melhoria da função gonadal; assim, a relação entre o excesso de peso e os níveis de T é bidirecional, configurando círculo vicioso.^21^ Neste estudo, as sub-análises de categorias de IMC mostraram que o aumento de peso normal para obeso acrescentou risco relativo de 32,1% de desenvolvimento de DT, enquanto que de sobrepeso para obeso acrescentou risco relativo de 18,9%. Além disso, o aumento de 1 kg/m^2^ no IMC gerou queda de 8,44 ng/dL na TT (B=-8,44; IC95%= -12,57 até -4,32; p<0,0001).

Existe um declínio natural dos hormônios sexuais masculinos de aproximadamente 1% ao ano com o envelhecimento, entre as idades de 40 e 70 anos.^22^ A prevalência de hipogonadismo de início tardio (" *Late onset hypogonadism* "), associa-se não só ao envelhecimento, como também com aumento o no IMC e nas doenças coexistentes.22 Nesse sentido, este estudo buscou correlacionar o avançar da idade com a diminuição dos níveis de TT. Contudo, o valor de p, embora marginal, não foi estatisticamente significante (p=0,0520). No entanto, a idade mostrou-se uma variável significativa para a queda de TT na análise multivariada realizada, sendo que o acréscimo de um ano na idade gerou decréscimo de 3,79 ng/dL na TT ( *B* =-3,79; IC95%=-5,57 até -2,06; p<0,0001). Tais achados possivelmente seriam mais eloquentes em uma amostra maior.

Svartberg et al.^7^ estudaram a pressão arterial em 1548 homens e os valores de TT foram inversamente associados à pressão arterial sistólica (p<0,001) e à massa ventricular esquerda (p<0,001). Porém, após o ajuste para o IMC, os resultados sugeriram que essa relação foi mediada pela obesidade.7 Zitzmann e Nieschlag^23^ encontraram relações semelhantes, sendo que a reposição de T resultou em decréscimos significativos desses parâmetros e da frequência cardíaca. Partindo desse racional, este estudo buscou associações entre os valores de TT e os fatores tempo de diagnóstico de HA (p=0,1418) e o número de classes de anti-hipertensivos usados (p=0,0732). Porém, os achados não apresentaram significância estatística, o que talvez se justifique pelo fato de os fatores aqui pesquisados, embora inéditos na literatura e indiretamente relacionados aos valores de pressão arterial propriamente ditos, difiram desses em última análise.

A espironolactona é um anti-hipertensivo com propriedade anti-androgênica por múltiplos mecanismos.^24^ Por isso, investigamos se o seu emprego no tratamento da HA poderia ser um fator de confusão ao se associar com a diminuição dos valores de TT, o que não foi confirmado ( *p* =0,3546) em nossa amostra.

O quadro clínico do DT pode ser negligenciado, pois muitas vezes simula queixas condizentes com o envelhecimento ou com outras comorbidades.^22^ O questionário de deficiência de andrógenos da Universidade de Saint Louis (ADAM)^13^ é amplamente utilizado como ferramenta de triagem para detectar homens em risco de deficiência androgênica.^16^ Neste estudo, porém, não foi observada correlação entre nível de TT e questionário ADAM positivo neste estudo (p=0,2483). Em nossa amostra, apenas 10% dos indivíduos apresentaram questionário ADAM negativo, o que possivelmente se justifica pelo fato de a HA estar associada à presença de queixas interrogadas no questionário, fazendo, assim, com que ele perdesse especificidade.

A disfunção testicular atinge cerca de 50% dos homens com DRC,^12^ com diferentes estágios,^24^ sendo associada ao aumento da mortalidade por todas as causas nessa população.^25^ Assim, nós avaliamos se a presença de DRC estaria associada à DT, o que não se confirmou neste estudo ( *p* =0,3321). Tal fato poderia estar relacionado ao fato de que a maioria (93,5%) dos pacientes com DRC deste estudo apresentaram taxa de filtração glomerular superior à 30 mL/min/1,72m^2^.

Os níveis séricos de T também se relacionam com o metabolismo lipídico, havendo relação inversa entre valores de T e triglicerídeos, colesterol total e colesterol LDL, enquanto bons níveis de T acompanham-se de HDL com valores normais.^26^ Schiffer et al.^27^ observaram que indivíduos expostos à terapia de privação androgênica apresentam quadro de DLP. Neste estudo, não observamos correlação estatisticamente significante entre a presença de DLP e DT (p=0,3388), o que talvez pode ser explicado pelo fato de a nossa amostra ser de pacientes intensamente tratados.

Aproximadamente um terço dos portadores de DM tipo 2 (DM2) tem baixos níveis de T.^28^ Oh et al.^29^ apontam a DT como um possível fator de risco ao desenvolvimento de DM2. Homens com valores altos de T apresentam risco 42% menor de DM2, indicando que níveis elevados do hormônio sejam fator de proteção ao desenvolvimento de DM2.^30^ Em nosso estudo, porém, a presença de DM não apresentou correlação significativa com DT ( *p* =0,1112). Isso pode ser reflexo da heterogeneidade da nossa população, com controle glicêmico e intensidade de tratamento variados, sendo que 19,5% dos pacientes com DM da amostra apresentavam classificação de pré-diabetes,^10^ e poucos pacientes em insulinoterapia.

A T é o principal hormônio sexual masculino^22^ e desempenha papel crucial na saúde reprodutiva, assim como na saúde geral e na qualidade de vida do indivíduo.15 As evidências atuais apoiam fortemente a premissa de que baixo nível de T é importante biomarcador de morbimortalidade em homens,^31^ sendo as consequências metabólicas mais adversas quanto mais baixa a T.^21^

Quando a T é produzida, passa a circular ligada à globulina de ligação a hormônio sexual (SHBG) e à albumina, sendo que apenas pequena fração da T, de aproximadamente 2%, circula livremente como "Testosterona Livre" (TL),15 que corresponde à sua forma ativa. As concentrações da SHBG variam em função de fatores como idade, obesidade e outras doenças crônicas.^32^

Esta pesquisa foi um estudo de vida real. Entre suas limitações consta o fato de os exames bioquímicos terem sido realizados de forma randômica e em sua maioria na rede pública de saúde e, por isso, não houve uniformidade entre os métodos laboratoriais empregados. Além disso, os valores de TL não terem sido avaliados, devido aos custos envolvidos com os exames que permitiriam seu cálculo indireto.^15^

Este é o primeiro estudo, ao nosso conhecimento, que buscou demonstrar a prevalência de DT em uma população especificamente hipertensa. O potencial deletério da deficiência androgênica é possivelmente inntensificado em um contexto de maior risco cardiovascular, o que nos alertaria sobre a importância de seu reconhecimento. A prevalência crescente da obesidade hoje reforça a necessidade de estratégias que promovam seu controle, sobretudo entre homens hipertensos, pois suas repercussões podem impactar em vários aspectos, dentre eles sua saúde sexual, fertilidade e qualidade de vida.

## Conclusões

O estudo encontrou alta prevalência de DT (26,8%) em pacientes hipertensos **,** sem, contudo, demonstrar associação entre nível de TT e tempo de diagnóstico de HA, número de classe de anti-hipertensivos, uso de espironolactona, sintomas positivos de DT, presença de DM, DLP, e DRC. Porém, houve forte associação inversa da TT com o IMC, sendo que o aumento do IMC em uma unidade se associou à queda em nos valores de TT em 8.44 ng/dL; assim como o avanço em um ano na idade associou-se ao seu declínio em 3,79 ng/dL. Novos estudos são necessários a fim de elucidar a possível associação entre a HA e DT.

## References

[1] Bhasin S, Brito JP, Cunningham GR, Hayes FJ, Hodis HN, Matsumoto AM (2018). Testosterone Therapy in Men with Hypogonadism: an Endocrine Society Clinical Practice Guideline. J Clin Endocrinol Metab.

[2] Feldman HA, Longcope C, Derby CA, Johannes CB, Araujo AB, Coviello AD (2002). Age Trends in the Level of Serum Testosterone and Other Hormones in Middle-Aged Men: Longitudinal Results from the Massachusetts Male Aging Study. J Clin Endocrinol Metab.

[3] Kelleher S, Conway AJ, Handelsman DJ (2004). Blood Testosterone Threshold for Androgen Deficiency Symptoms. J Clin Endocrinol Metab.

[4] Bhasin S, Cunningham GR, Hayes FJ, Matsumoto AM, Snyder PJ, Swerdloff RS (2010). Testosterone Therapy in Men with Androgen Deficiency Syndromes: an Endocrine Society Clinical Practice Guideline. J Clin Endocrinol Metab.

[5] Shores MM, Matsumoto AM, Sloan KL, Kivlahan DR (2006). Low Serum Testosterone and Mortality in Male Veterans. Arch Intern Med.

[6] Mulligan T, Frick MF, Zuraw QC, Stemhagen A, McWhirter C. (2006). Prevalence of Hypogonadism in Males Aged at Least 45 Years: the HIM Study. Int J Clin Pract.

[7] Svartberg J, von Mühlen D, Schirmer H, Barrett-Connor E, Sundfjord J, Jorde R. (2004). Association of Endogenous Testosterone with Blood Pressure and Left Ventricular Mass in Men. The Tromsø Study. Eur J Endocrinol.

[8] Smith MR, Lee H, Nathan DM (2006). Insulin Sensitivity During Combined Androgen Blockade for Prostate Cancer. J Clin Endocrinol Metab.

[9] (2016). Associação Brasileira para o Estudo da Obesidade e da Síndrome Metabólica. Diretrizes Brasileiras de Obesidade.

[10] Sociedade Brasileira de Diabetes (2019). Diretrizes da Sociedade Brasileira de Diabetes 2019-2020.

[11] Faludi AA, Izar MC, Saraiva JF, Chacra AP, Bianco HT, Afiune A (2017). Atualização da Diretriz Brasileira de Dislipidemias e Prevenção da Aterosclerose – 2017. Arq Bras Cardiol.

[12] Willis K, Cheung M, Slifer S (2013). International Society of Nephrology. KDIGO 2012 Clinical Practice Guideline for the Evaluation and Management of Chronic Kidney Disease. Kidney Int Suppl.

[13] Morley JE, Charlton E, Patrick P, Kaiser FE, Cadeau P, McCready D (2000). Validation of a Screening Questionnaire for Androgen Deficiency in Aging Males. Metabolism.

[14] Mulhall JP, Trost LW, Brannigan RE, Kurtz EG, Redmon JB, Chiles KA (2018). Evaluation and Management of Testosterone Deficiency: AUA Guideline. J Urol.

[15] Kanakis GA, Tsametis CP, Goulis DG (2019). Measuring Testosterone in Women and Men. Maturitas.

[16] Mohamed O, Freundlich RE, Dakik HK, Grober ED, Najari B, Lipshultz LI (2010). The Quantitative ADAM Questionnaire: a New Tool in Quantifying the Severity of Hypogonadism. Int J Impot Res.

[17] Sociedade Brasileira de Endocrinologia, Sociedade Brasileira de Urologia (2017). Hipogonadismo Masculino Tardio ou Deficiência Androgênica do Envelhecimento Masculino (DAEM): diagnóstico.

[18] Field A. (2009). Discovering Statistics with SPSS.

[19] Grossmann M. (2018). Hypogonadism and Male Obesity: Focus on Unresolved Questions. Clin Endocrinol.

[20] Lamm S, Chidakel A, Bansal R. (2016). Obesity and Hypogonadism. Urol Clin North Am.

[21] Carrageta DF, Oliveira PF, Alves MG, Monteiro MP (2019). Obesity and Male Hypogonadism: Tales of a Vicious Cycle. Obes Rev.

[22] Hochreiter WW, Ackermann DK, Brütsch HP (2005). Andropause. Ther Umsch.

[23] Zitzmann M, Nieschlag E. (2007). Androgen Receptor Gene Cag Repeat Length and Body Mass Index Modulate the Safety of Long-Term Intramuscular Testosterone Undecanoate Therapy in Hypogonadal Men. J Clin Endocrinol Metab.

[24] Bagnoli VR, Fonseca AM, Cezarino PY, Fassolas G, Arie JA, Baracat EC (2010). Tratamento Hormonal da Acne Baseado em Evidências. Femina.

[25] Khurana KK, Navaneethan SD, Arrigain S, Schold JD, Nally JV, Shoskes DA (2014). Serum Testosterone Levels and Mortality in Men with CKD Stages 3-4. Am J Kidney Dis.

[26] Rastrelli G, Filippi S, Sforza A, Maggi M, Corona G. (2018). Metabolic Syndrome in Male Hypogonadism. Front Horm Res.

[27] Schiffer L, Kempegowda P, Arlt W, O'Reilly MW (2017). Mechanisms in Endocrinology: The Sexually Dimorphic Role of Androgens in Human Metabolic Disease. Eur J Endocrinol.

[28] Dhindsa S, Prabhakar S, Sethi M, Bandyopadhyay A, Chaudhuri A, Dandona P. (2004). Frequent Occurrence of Hypogonadotropic Hypogonadism in Type 2 Diabetes. J Clin Endocrinol Metab.

[29] Oh JY, Barrett-Connor E, Wedick NM, Wingard DL (2002). Endogenous Sex Hormones and the Development of Type 2 Diabetes in Older Men and Women: the Rancho Bernardo Study. Diabetes Care.

[30] Ding EL, Song Y, Malik VS, Liu S. (2006). Sex Differences of Endogenous Sex Hormones and Risk of Type 2 Diabetes: a Systematic Review and Meta-Analysis. JAMA.

[31] Zarotsky V, Huang MY, Carman W, Morgentaler A, Singhal PK, Coffin D (2014). Systematic Literature Review of the Risk Factors, Comorbidities, and Consequences of Hypogonadism in Men. Andrology.

[32] Bahia L, Dimetz T, Gazolla H, Clemente E, Gomes MB (2000). Interrelações entre SHBG e Esteróides Sexuais com Medidas Antropométricas, Pressão Arterial e Lipídeos em Mulheres com e sem Diabetes Mellitus tipo 2. Arq Bras Endocrinol Metab.

