# Supplementary material for: Deficiência de Testosterona em Homens Hipertensos: Prevalência e Fatores Associados
Source: Arq Bras Cardiol. 2024 Mar 21;121(3):e20230138. [Article in Portuguese] doi: 10.36660/abc.20230138 (PMC12094286; doi:10.36660/abc.20230138)
Supplement: Translation

# Translation: Testosterone Deficiency in Hypertensive Men: Prevalence and Associated Factors

**DOI:** 10.36660/abc.20230138i

**Authors:** Leandra Analia Freitas Negretto, Nelson Rassi, Leonardo Ribeiro Soares, Amanda Bueno Carvalho Saraiva, Maria Emília Figueiredo Teixeira, Luciana da Ressurreição Santos, Ana Luiza Lima Souza, Paulo Cesar B. Veiga Jardim, Weimar Kunz Sebba Barroso de Souza, Thiago de Souza Veiga Jardim

**Affiliations:** 1 Goiânia GO Brazil Universidade Federal de Goiás – Liga de Hipertensão Arterial, Goiânia, GO – Brazil; 2 Goiânia GO Brazil Hospital Geral de Goiânia – Dr. Alberto Rassi, Goiânia, GO – Brazil

**Keywords:** Testosterone, Hypertension, Obesity, Hypogonadism

## Abstract

**Background:**

Testosterone deficiency (TD) is a prevalent condition in our midst and still very neglected. Arterial hypertension (AH) is one of the possible associated factors.

**Objectives:**

To determine the prevalence of TD in a hypertensive male population and the factors associated with its occurrence, such as age, time since hypertension diagnosis, number of antihypertensive classes, body mass index (BMI), diabetes, dyslipidemia, chronic kidney disease (CKD), positive symptoms of TD (positive ADAM questionnaire) and use of spironolactone.

**Methods:**

Cross-sectional study with administration of the ADAM questionnaire, assessment of biochemical, clinical, and anthropometric data. Patients were stratified into DT and normal testosterone groups. Categorical variables were compared using the chi-squared test and continuous variables using the Mann-Witney test; variables with significance (p<0,05) were analyzed by multivariable linear regression.

**Results:**

The prevalence of TD was 26.36%. There was an association between TD and body mass index (BMI) (p=0.0007) but there was no association with age (p=0.0520), time of hypertension diagnosis (p=0.1418), number of classes of antihypertensive drugs (p=0.732), diabetes (p=0.1112); dyslipidemia (p=0.3888); CKD (p=0.3321); use of spironolactone (p=0.3546) or positive ADAM questionnaire (p=0.2483).

**Conclusions:**

TD was highly prevalent and positively associated with BMI. Total testosterone (TT) declined by 8.44ng/dL with a one unit increase in BMI and dropped by 3.79ng/dL with a one-year increase in age.

## Introduction

Testosterone deficiency (TD) results from failure of the testes to produce physiological levels of testosterone (T) due to rupture in at least one of the elements of the hypothalamic-pituitary-testicular (HPT) axis. When TD is associated with clinical signs of androgen deficiency, the diagnosis of male hypogonadism may be established.^1^The prevalence of hypogonadism in male population varies with age, ranging from six to 12.3% between 40 and 69 years of age, respectively.^2^

TD may cause different signs and symptoms that depend on age of onset and TD severity,^3^and include: reduced libido, erectile dysfunction, loss of body and facial hair, loss of lean mass, increase of fat mass, fatigue and anemia.^3 , 4^In addition, survival of men with low T levels is lower than of men with normal T levels (eugonadism), with an all-cause mortality of 34.9% ***vs.*** 20.1%, respectively.^5^

Hypertension has been associated with TD in previous studies, as in the "Hypogonadism in males" (HIM),^6^which showed that a higher proportion of hypogonadal patients was hypertensive as compared with eugonadal subjects. According to Svartberg et al.,^7^hypertensive individuals have lower baseline T levels than nonhypertensive ones, regardless of age. Smith et al.^8^showed that androgen deprivation therapy for prostate cancer could induce hypertension.

The relationship between hypertension and TD has many gaps, including epidemiological, diagnostic, and therapeutic issues. In this context, the aim of this study was to determine the prevalence of TD in a cohort of hypertensive men seen at a referral hypertension center in Brazil. Also, the study evaluated, in this population, factors related to its occurrence – age, time of diagnosis of hypertension, number of antihypertensive classes, body mass index (BMI),^9^presence of diabetes mellitus (DM),^10^dyslipidemia (DLP),^11^chronic kidney disease (CKD),^12^positive symptoms of androgenic deficiency (ADAM, ***Androgen Deficiency, in the Aging Male*** questionnaire),^13^and use of spironolactone.

## Methods

### Study design

This was a cross-sectional study conducted in a referral hypertension center located in the Midwest of Brazil. Data were collected by the assistant staff during the outpatient visits.

### Patients

Male patients aged from 18 to 85 years old, who attended at least one visit a year were included. Patients who did not perform the tests required, transsexual patients, and patients with cognitive impairment who were not able to answer the ADAM questionnaire were excluded.^13^

Sample size calculation was carried out using the G*Power 3.1, with an expected prevalence of 12%,^2^confidence interval (CI) of 95%, level of significance 0.05 and 80% test power, resulting in a minimum of 148 individuals.

### Data collection procedures

In this study, the following variables were considered for analysis: age, time of diagnosis of hypertension, number of antihypertensive classes, use of spironolactone, BMI,^9^presence of DM,^10^DLP,^11^CKD,^12^and positive symptoms of androgen deficiency (ADAM questionnaire^13^).

During the outpatient visits, after the patients signed the informed consent form, they had their weight and height measured, the abovementioned variables assessed, and laboratory tests, including total T (TT) measurements, were ordered. A TT cut-off of 300ng/dL (12nmol/L) was used to define TD, following the Endocrine Society^1^and the American Urological Association^14^recommendations. Blood samples were collected in fasting state in the morning and the TT levels were determined in randomized laboratories where the radioimmunoassay was available.^15^

The ADAM questionnaire, from the Saint Louis University, consists of 10 "yes" or "no" questions about androgen deficiency symptoms.^13^This questionnaire has an 88% sensitivity and a 60% specificity,^16^and is recommended as a screening tool for hypogonadism.^17^

### Statistical analysis

A total of 239 participants were assessed and stratified into two groups according to TT status – individuals with TD ( ***n*** =64; 26.8%) and (2) eugonadal men ( ***n*** =175; 73.2%). In descriptive statistics, categorical variables were described as absolute (n) and relative frequencies [ *f(%)* ]; and continuous variables were described as median and interquartile range (IQR). The categorical variables were TT status, age, time of hypertension, number of antihypertensive classes, BMI, DM DLP, CKD, use of spironolactone and ADAM questionnaire. Continuous quantitative variables were age, time of hypertension, number of antihypertensive classes, BMI and TT levels (ng/dL).

In the inferential statistics analysis, categorical variables were stratified by TT status and compared using the Fisher's exact test or the G's test. For statistically significant variables, the odds ratio (OR) and 95% CI were calculated to evaluate the odds of TD between the groups.

For continuous variables, data normality was calculated using the Kolmogorov–Smirnov test, and variance homogeneity using the Levene's test. All continuous variables had a non-normal, non-homogeneous distribution (p<0.05) and, for this reason, the Mann-Whitney test for independent samples was used. Then, variables with a p<0.20 were analyzed using a multivariate linear regression model. Finally, the Spearman correlation was also used.^18^

## Results

A total of 276 hypertensive men were recruited; 37 were excluded – 18 were older than 85 years, 11 declined to participate in the study, three had cognitive impairment, two had schizophrenia, two died before blood collection and one was transsexual. Among the 239 patients included, 175 (73.2%) had normal TT levels (eugonadism), yielding a TD prevalence of 26.8%. The results are summarized in the Central Illustration.

Table 1 describes TT status ( ***i.e*** ., TD or eugonadal, age), time of hypertension diagnosis, number of antihypertensive classes, BMI,^9^DM,^10^DLP,^11^and CKD,^12^use of spironolactone and the ADAM questionnaire results.^13^These data are expressed as absolute (n) and relative frequencies [ *f(%)* ].

**Table 1:** Descriptive statistics of categorical variables, expressed as absolute (n) and relative frequencies [ *f(%)* ], of 239 patients, Goiania, Brazil, 2023

Variables (N=239)			*n*	*f(%)*
**Testosterone status**				
	Testosterone deficiency		64	26.8
	Eugonadal		175	73.2
**Age (years)**				
	< 60 years		91	38.1
	≥ 60 years		148	61.9
**Time of hypertension (years)**				
	< 15 years		126	52.7
	≥ 15 years		97	40.6
	Not informed		16	6.7
**Number of antihypertensive classes**				
	None		8	3.3
	<3		184	77.0
	>3		47	19.7
**BMI (Kg/m^2^)**				
	Normal (18.6 - 24.9)		51	21.3
	Overweight (25.0 - 29.9)		100	41.8
	Obesity (≥30.0)			
		Grade I (30.0 - 34.9)	58	24.3
		Grade II (35.0 - 39.0)	20	8.4
		Grade III (≥40.0)	8	3.3
	Not informed		2	0.8
**Diabetes**				
	Yes		78	32.6
	No		161	67.4
**Dyslipidemia**				
	Yes		172	72.0
	No		67	28.0
**Chronic kidney disease**				
	Yes		78	32.6
	No		161	67.4
**Spironolactone**				
	Yes		23	9.6
	No		216	90.4
**ADAM questionnaire**				
	Negative		24	10.0
	Positive		184	77.0
	Not informed		31	13.0

N: total number of individuals in the sample; n: absolute frequency; f(%): relative frequency; BMI: body mass index; ADAM: Androgen Deficiency, in the Aging Male.

Table 2 compares the variables and respective categories between TD and eugonadal patients. There was a statistically significant difference in BMI (p=0.0007) between the groups, with a greater number of obese patients in the TD group.

**Table 2:** Inferential statistics of categorical variables of 239 participants, stratified by testosterone status; Goiania, Brazil, 2023

Variables (N=239)			Testosterone deficiency (n=64; 26.8%)		Eugonadal (n=175; 73,2%)		p-value *
			*n*	*f(%)*	*n*	*f(%)*	
**Age (years)**							
	< 60 years		18	28.1	73	41.7	
	≥ 60 years		46	71.9	102	58.3	0.0520
**Time of hypertension (years)**							
	< 15 years		28	43.8	98	56.0	
	≥ 15 years		30	46.9	67	38.3	0.1418
	Not informed		6	9.4	10	5.7	
**Number of antihypertensive classes**						
	None		0	0.0	8	4.6	
	< 3		52	81.3	132	75.4	
	>3		12	18.8	35	20.0	0.0732
**BMI (Kg/m^2^)**							
	Normal (18.6 - 24.9)		5	7.8	46	26.3	
	Overweight (25.0 - 29.9)		23	35.9	77	44.0	
	Obesity (≥30.0)						
		Grade I (30.0 - 34.9)	24	37.5	34	19.4	
		Grade II (35.0 - 39.0)	9	14.1	11	6.3	
		Grade III (≥40.0)	3	4.7	5	2.9	**0.0007**
	Not informed		0	0.0	2	1.1	
**Diabetes**							
	Yes		26	40.6	52	29.7	
	No		38	59.4	123	70.3	0.1112
**Dyslipidemia**							
	Yes		49	76.6	123	70.3	
	No		15	23.4	52	29.7	0.3388
**Chronic kidney disease**							
	Yes		24	37.5	54	30.9	
	No		40	62.5	121	69.1	0.3321
**Spironolactone**							
	Yes		8	12.5	15	8.6	
	No		56	87.5	161	92.0	0.3546
**ADAM questionnaire**							
	Negative		4	6.3	20	11.4	
	Positive		51	79.7	133	76.0	0.2483
	Not informed		9	14.1	22	12.6	

N: total number of individuals in the sample; n: absolute frequency; f(%): relative frequency; BMI: body mass index; ADAM: Androgen Deficiency, in the Aging Male;

* chi-square test, Fisher's exact test or the G test.

There was a predominance of obese individuals in the TD group, BMI ( *χ* ^2^=18,10; p=0.0001). For comparison between BMI categories, there were statistically significant differences between: normal BMI vs. obese (OR=6.62; 95%CI=2.39–18.32; p=0.0002), with increment of relative risk of 32.1; overweight vs. obese (OR=2.41; 95%CI=1.28–4.54; p=0.0094), with increment of relative risk of 18.9%. Table 3 shows statistically significant differences between the TD and eugonadal groups in the time of hypertension (p=0.317), BMI (p<0.0001) and TT (p<0.0001).

**Table 3:** Median and interquartile range (IQR) of continuous quantitative variables of 239 patients, stratified and compared by testosterone status; Goiania, Brazil, 2023

Variables	DT (n=64; 26.8%)			Eugonadal (n=175; 73.2%)			p-valor *	Total (N=239)		
	*Mediana*	*IQR*		*Mediana*	*IQR*			*Mediana*	*IQR*	
		25%	75%		25%	75%			25%	75%
Age (years)	63.5	58.8	70.0	62.0	55.0	70.5	0.3476	63.0	55.5	70.0
Time of hypertension (years)	**16.5**	8.0	24.3	**14.0**	6.0	20.0	**0.0317**	15.0	7.0	20.0
Number of antihypertensives	3.0	2.0	3.0	3.0	2.0	3.0	0.7242	3.0	2.0	3.0
BMI (Kg/m^2^)	**31.0**	27.3	34.5	**27.7**	24.9	30.8	**<0.0001**	28.5	25.4	31.6
Total testosterone (ng/dL)	**220.0**	182.5	262.8	**449.0**	360.0	532.0	**<0.0001**	376.7	286.3	491.5

TD: Testosterone deficiency; N: total number of individuals in the sample; Median: measure of central tendency; IQR: interquartile range (25% - 75%); BMI: body mass index.

* Mann-Whitney test for independent samples

**Table 4:** Spearman correlation between continuous quantitative variables; Goiania, Brazil, 2023

Variables		Testosterone (ng/dL)	Age (years)	SAH (years)	Antihypertensives	BMI (Kg/m^2^)
Testosterone	ρ (rho)	1.0000				
	*p-valor*					
Age (years)	ρ (rho)	**-0.1760**	1.0000			
	*p-valor*	**0.0068**				
SAH (years)	ρ (rho)	**-0.1410**	**0.3530**	1.0000		
	*p-valor*	**0.0366**	**<0.0001**			
Antihypertensives	ρ (rho)	-0.1046	**0.1700**	**0.2640**	1.0000	
	*p-valor*	0.1083	**0.0083**	**0.0001**		
BMI (kg/m^2^)	ρ (rho)	**-0.2600**	**-0.2330**	-0.0048	0.0521	1.0000
	*p-valor*	**0.0001**	**0.0003**	0.9434	0.4248	

SAH: systemic arterial hypertension; BMI: body mass index.

The Spearman correlation between continuous quantitative variables revealed a low, inversely proportional and statistically significant relationship between TT and BMI (ρ=-0.26; p=0.0001), age (ρ=-0.1760; p=0.0069) and time of hypertension diagnosis (ρ=-0.1410; p=0.0366).

The variables that showed statistically significant difference are shown in Figure 1 : (a) TT; (b) BMI and (c) time of diagnosis of HA. Patients with TD showed median TT of 222.0 ng/dL, BMI of 31.0 Kg/m^2^and 16.5 years of diagnosis of HA, whereas the eugonadal subjects showed median TT of 449.0 ng/dL, BMI of 27.7 Kg/m^2^and 14.0 years of diagnosis of HA.

**Figure 1:** Box plots with median, interquartile range (25% and 75%), minimum and maximum values of variables that showed statistically significant difference: (a) total testosterone; (b) Body mass index and (c) time of diagnosis of hypertension; Goiania, Brazil.

A multivariate linear regression analysis of variables with low p-values (p<0.20) was performed, since all statistical assumptions (linearity, independency, homoscedasticity, normality of residuals, non-collinearity and lack of outliers) were met. The variables that had the strongest influence on TT were BMI and age. A one-year increase in age was associated with a 3.79ng/dL decrease in TT (B=-3.79; 95%CI=-5.57 to -2.06; p<0.0001), and a one unit increase in BMI was associated with a 8.44 ng/dL decrease in TT (B=-8.44; 95%CI= -12.57 to -4.32; p<0.0001) ( Figure 2 ).

**Figure 2:** Relationship between total testosterone (ng/dL) and body mass index (BMI, Kg/m^2^) of all participants (N=239).

## Discussion

To our knowledge, this is the first study to evaluate the prevalence of TD in a hypertensive population. We found a 25.8% prevalence of TD, and an association between TD and BMI (p=0.0007).

Obesity is the clinical condition with the strongest relationship with decreased TT levels in men.^19 , 20^Weight excess causes HPT axis suppression in a functional and potentially reversible manner.^19^Low T levels lead to an increase in adiposity due to the lack of inhibition of adipogenesis and lipid uptake, mediated by the lipoprotein lipase enzyme, mainly in abdominal fat deposits.^21^On the other hand, weight loss can lead to amelioration of gonadal function; therefore, the relationship between weight excess and T levels is bidirectional, establishing a vicious cycle.^21^In the present study, analysis of the BMI categories showed that the increase in BMI from normal weight to obesity led to an increase in relative risk of 18.9%. Besides, a one Kg/m^2^increase in BMI was associated with an 8.44 ng/dL decrease in TT (B=-8.44; 95%CI= -12.57 to -4.32; p<0.0001).

There is a natural decline of approximately 1% a year in male sexual hormones with aging between 40 and 70 years of age.^22^The prevalence of late onset hypogonadism is not only associated with aging but also with the increase in BMI and coexisting diseases.^22^In this regard, our study aimed to correlate the advance in age with the decrease in TT levels. However, despite marginal, the p-value was not statistically significant (p=0.0520). Age, in turn, has shown to be a significant variable in the multivariate analysis, and a one-year increase in age was associated with a 3.79ng/dL decrease in TT ( *B* =-3.79; 95%CI=-5.57 to -2.06; p<0.0001). Probably, these findings would be more expressive in a larger sample.

Svartberg et al.^7^assessed blood pressure in 1,578 men, and TT values were inversely related to systolic blood pressure (p<0.001) and left ventricular mass (p<0.001). However, after adjustments for BMI, the results suggested that this relationship was mediated by obesity.^7^Similar results were found by Zitzmann & Nieschlag,^23^and T reposition resulted in significant decrements of these parameters and heart rate. Based on this rational, the present study evaluated associations of TT values with the time of hypertension diagnosis (p=0.1418) and the number of antihypertensive classes used by the patients (p=0.0732). However, the findings were not statistically significant, which may be justified by the fact that the factors investigated, although published for the first time and indirectly related to blood pressure measurements, ultimately differ from them.

Spironolactone is an antihypertensive agent that has antiandrogenic properties due to multiple mechanisms.^24^Thus, we investigated whether its use in the treatment of hypertension would be a confound factor for its association with TT values, which was not confirmed in our sample (p=0.3546).

Clinical presentation of TD may be easily neglected, since the symptoms are common to those of aging and other comorbidities.^22^The Saint Louis University ADAM questionnaire^13^has been widely used as a screening tool for men at risk of androgen deficiency.^16^In the present study, we did not observe any correlation between TT levels and positive ADAM questionnaire. In our sample, there was only 10% of patients with a negative questionnaire, which may be explained by the fact that hypertension *per se* is associated with the complaints assessed by the questionnaire, hence affecting the specificity of the instrument.

Testicular dysfunction affects nearly 50% of men with CKD,^12^in different stages,^24^and is associated with an increase in all-cause mortality in this population.^25^Therefore, we investigated whether CKD was associated with TD, which was not confirmed in this study ( *p* =0.3321). This could be explained by the fact that most (93.5%) of our patients with CKD had a glomerular filtration rate greater than 30 mL/min/1.72m^2^.

In addition, serum T levels are also associated with lipid metabolism, with an inverse relationship between T values and triglycerides, total cholesterol and LDL cholesterol, whereas good T levels are associated with normal HDL levels.^26^Schiffer et al.^27^observed that patients receiving androgen deprivation therapy developed DLP. In the present study, we did not observe a statistically significant correlation between the presence of DLP and TD (p=0.3388), which may be explained by the fact that our sample consisted of intensely treated patients.

Nearly one third of patients with type 2 DM (DM2) has low T levels.^28^Oh et al.^29^suggested that TD is a potential risk factor for DM2. Men with high T levels have a 42% lower risk of DM2, indicating that high levels of the hormone are protective against the disease.^30^However, in our study, the presence of DM did not show a significant correlation with TD (p=0.112). This may be due to the heterogeneity of our study population, with different glycemic control and treatment intensity; 19.5% of the DM patients were prediabetic,^10^and few patients were on insulin therapy.

T is the main male sexual hormone,^22^and it plays a crucial role in reproductive function, as well as in individuals’ general health and quality of life.^15^Current evidence strongly supports the premise that low T levels is an important biomarker of morbidity and mortality in men,^31^with patients with lower T levels experiencing more severe metabolic consequences.^21^

After T is produced, it circulates bound to either sex hormone binding globulin (SHBG) or albumin, and only a small fraction circulates as free testosterone,^15^which is its active form. Concentrations of SHBG vary with age, and presence of chronic diseases such as obesity.^32^

This was a real-world study; one of its limitations is the fact that laboratory tests were performed randomly, mostly in the public sector and, for this reason, the biochemical methods were not uniform. Besides, free testosterone levels were not assessed due to high costs of the tests that would enable its indirect estimation.^15^

As far as we know, this is the first study to determine the prevalence of TD in a hypertensive population. Potential deleterious effects of androgen deficiency are possibly intensified in this context of increased cardiovascular risk, which draws attention to the importance of the identification of this condition. The growing prevalence of obesity today reinforces the need for strategies that promote its control, especially in hypertensive men, since its repercussions may impact different aspects of their lives, including sexual life, fertility, and quality of life.

## Conclusions

The present study found a high prevalence of TD (26.8%) in hypertensive patients, but no association of TT levels with the time of diagnosis of hypertension, number of antihypertensive classes, use of spironolactone, positive symptoms of TD, presence of DM, DLP, or CKD was found. On the other hand, there was a strong, inverse relationship between TT and BMI, and one unit increase in BMI was associated with an 8.44 ng/dL decrease in TT. Also, an increment of one year old was associated with a decrease of 3.79 ng/dL in TT concentrations. Further studies are needed to elucidate the possible association between hypertension and TD.
